# Lung epithelial response to cigarette smoke and modulation by the nicotinic alpha 7 receptor

**DOI:** 10.1371/journal.pone.0187773

**Published:** 2017-11-08

**Authors:** Lorise C. Gahring, Elizabeth J. Myers, Diane M. Dunn, Robert B. Weiss, Scott W. Rogers

**Affiliations:** 1 Geriatric Research, Education, and Clinical Center, Salt Lake City Veterans Administration Medical Center, Salt Lake City, Utah, United States of America; 2 Division of Geriatrics, Department of Internal Medicine, University of Utah School of Medicine, Salt Lake City, Utah, United States of America; 3 Department of Human Genetics, University of Utah School of Medicine, Salt Lake City, Utah, United States of America; 4 Department of Neurobiology and Anatomy, University of Utah School of Medicine, Salt Lake City, Utah, United States of America; Emory University School of Medicine, UNITED STATES

## Abstract

Cigarette smoking (CS) is a principal contributor to a spectrum of devastating lung diseases whose occurrence and severity may vary between individuals and not appear for decades after prolonged use. One explanation for the variability and delay in disease onset is that nicotine, the addictive component of CS, acts through the ionotropic nicotinic acetylcholine receptor (nAChR) alpha7 (α7) to modulate anti-inflammatory protection. In this study we measured the impact α7 signaling has on the mouse distal lung response to side-stream CS exposure for mice of the control genotype (α7^G^) and those in which the α7-receptor signaling mechanisms are restricted by point mutation (α7^E260A:G^). Flow cytometry results show that after CS there is an increase in a subset of CD11c (CD11c^hi^) alveolar macrophages (AMs) and histology reveals an increase in these cells within the alveolar space in both genotypes although the α7^E260A:G^ AMs tend to accumulate into large aggregates rather than more widely distributed solitary cells common to the α7^G^ lung after CS. Changes to lung morphology with CS in both genotypes included increased tissue cavitation due to alveolar expansion and bronchial epithelium dysplasia in part associated with altered club cell morphology. RNA-Seq analysis revealed changes in epithelium gene expression after CS are largely independent of the α7-genotype. However, the α7^E260A:G^ genotype did reveal some unique variations to transcript expression of gene sets associated with immune responsiveness and macrophage recruitment, hypoxia, genes encoding mitochondrial respiration complex I and extracellular fibrillary matrix proteins (including alterations to fibrotic deposits in the α7^G^ proximal airway bronchioles after CS). These results suggest α7 has a central role in modulating the response to chronic CS that could include altering susceptibility to associated lung diseases including fibrosis and cancer.

## Introduction

Tobacco cigarette smoking (CS) is well established as a principal contributor to a spectrum of devastating lung diseases. CS delivers a chronic pro-inflammatory challenge by particulates and irritants to the lungs, yet despite repetitive challenge, crippling complications to the user may not appear for decades after initiation of use. One possible reason to explain this is that nicotine itself has anti-inflammatory properties that may counteract the impact of the other CS agents, at least in part, as described by the “cholinergic anti-inflammatory pathway” [1–3]. The majority of nicotine’s modulatory activity on inflammation is imparted through its interaction with the ionotropic nicotinic acetylcholine receptor (nAChR) alpha7 (α7) whose extraordinary calcium current is sufficient to modify the activity of Jak/Stat, NF-κB, Creb and other cellular signaling cascades [2–6]. This also means that the nicotine-α7 interaction imparts several parallel and potentially very different effects depending upon the target tissues and cells as well as the challenging agent (inflammagen). In the lung the cell specific expression of α7 includes neuronal cells (e.g., autonomic nervous system) as well as non-neuronal cells (including alveolar macrophages (AM), club cells and Type II alveolar cells [7]). The anti-inflammatory impact role of α7 has been best characterized in terms of the response to inflammagens such as lipopolysaccharide (LPS). In this case α7 tends to suppress the inflammatory responses acting through the vagal nerve as well as directly inhibiting macrophage pro-inflammatory NF-κB signaling and activation of cytokine cascades (e.g., TNFα; [2, 3, 8]). Additionally, effects by α7 on lung epithelial cells include modifying their response to LPS [7] through alterations to subsequent signaling processes leading to recruitment and infiltration of bone marrow cells into the lung as well as changes to local production of certain mucins (e.g., Muc5b), surfactant proteins and genes of fibrosis. An important issue that has been raised by these studies is what role does α7 have in modulating inflammatory processes in response to CS and whether or not these are distinct from those activated by LPS.

To elucidate the mechanistic role of the α7 receptor in inflammatory processes, we developed a mouse model [7, 9–12] in which a point mutation was introduced to limit α7 calcium coupling to cell signaling pathways (termed α7^E260A:G^) that when compared to the control mouse (α7^G^) provides a method to discriminate how α7 signaling in different cells types modifies the host response to an inflammatory challenge (for details see [7, 11, 12]). For example, the α7^E260A:G^ mouse lung exhibits a reduced response to LPS due in part both to the reduced inflammatory response by alveolar macrophages (AM; relative to control α7^G^ mice) and a decrease in the transcriptional response by epithelial cells that includes reduced signaling for recruitment of cells from the bone-marrow into the lung. Further, altered club cell morphology, reduced ciliated cell numbers, changes to constitutive mucin production and its accumulation in distal bronchial passages, and a marked increase in pro-fibrotic transcripts and associated deposits seen around proximal bronchia in the α7^E260A:G^ lung have been reported [7]. In this study we have extended our analysis to assess the role of α7 in modulating inflammatory responses induced by chronic CS. Results of flow cytometry show that after CS CD11c^+^/SiglecF^+^ AMs exhibit an increased CD11c^hi^ phenotype that is more robust in the α7^E260A:G^ mouse compared to the α7^G^, which is opposite to the effects of α7 following acute LPS administration. Lung histology demonstrates the accumulation of immune cells, often large aggregates, within the α7^E260A:G^ alveolar spaces that was not observed in the α7^G^ lung. There were also CS-associated changes in the control α7^G^ bronchial dysplasia that was associated with alterations to club cell morphology. Notably, similar differences in club cell morphology are already present in the α7^E260A:G^ mouse before CS exposure and these did not worsen after CS to the same magnitude as was measured in the control α7^G^ mice. The overall impact by CS on epithelial cell gene expression related to cell signaling as determined using RNA-Seq analysis was largely independent of α7-genotype although key exceptions to this, including altered immune profiles, differences in genes encoding mitochondrial respiration complex I and extracellular matrix proteins, were present. These results support the impact of α7 on different cell responses to be cell type specific and it varies according to the inflammagen challenge (i.e., acute LPS versus chronic CS). Further, normal α7-signaling is important to retention of healthy lung morphology and its absence, as could occur if this receptor is desensitized or inactivated by chronic ligand exposure ([4, 6, 13]), can alter the severity or predisposition to lung pathologies that associate with CS exposure.

## Material and methods

### Animals

Animals were maintained according to the Guide for the Care and use of Laboratory Animals of the National Institutes of Health and in accordance with protocols approved in advance by the Institutional Animal Care and Use Committee at the University of Utah (Protocol Number #12–06001) and the VA Medical Center (Protocol A14/17), Salt Lake City, UT. The construction and characterization of the α7^G^ and α7^E260A:G^ mouse lines has been reported [7, 9–12]. Briefly, α7 transcript expression is reliably reported through the co-expression of a bi-cistronic IRES-tauGFP reporter cassette introduced using the precision of homologous recombination into the 3’ end of the *Chrna7* gene (α7G). The α7^E260A:G^ point mutation was introduced into the α7^G^ background also using homologous recombination [12] to assure matched controls. This mutation in the homozygous receptor diminishes the calcium current by more than 90 percent (for discussion see [12]) and produces multiple phenotypes that include both developmental (e.g. [12]) and alterations to inflammatory cell response and recruitment that involves both direct modifications to immune cell signaling as well as signaling by cells expressing α7 in peripheral organs. Groups of age, gender and strain matched mice were housed as 4 or 5 animals per cage for each experiment.

### Cigarette smoking

Both α7^G^ and α7^E260A:G^ mice were exposed to side-stream cigarette smoke (CS; 5 days/week for 4 months) simultaneously using the Teague smoking chamber and reference cigarettes (3RF4 type; University of Kentucky Agricultural Research program). Control mice were exposed to room air. This was standardized to total suspended particulates (TSP) of 150 mg/m^3 that required 25–50 cigarettes per session beginning at 8:00 AM for approximately 225 minutes. Following CS exposure, blood was collected from each animal (N = 8–16 for each group) at harvesting and the serum tested with a commercially available ELISA (Calbiotech) for cotinine levels. In CS exposed mouse serum of the different α7 genotypes were not significantly different (CS animals (ng/ml): α7^G^ = 12.9 +/- 3.3; α7^E260A:G^ = 11.4 +/- 2.0; No CS, cotinine was not detected). The mice were used 24 hours after their last exposure. All experiments with CS exposed mice have been repeated at least twice with groups of 4 to 5 mice in each experimental condition (CS vs Control).

### Cell enrichment and flow cytometry

Methods of Flow cytometry and the collection of BALF have been described in detail previously [7, 11, 12]. After a lethal dose of tribromoethanol (Avertin) bronchial alveolar lavage fluid (BALF) was collected via insertion of a butterfly needle into the trachea and repeated gentle flushing of lungs with 1 ml of buffer (DPBS, 2% BSA, 0.05% EDTA). Following removal of BALF cells, the lung tissue (minus trachea and esophagus) was diced and incubated at 37°C for 40 min in dissociation medium (10 ml DMEM, 500 μl DNase 1 (1 mg/ml) containing 100 μl of 2.5 mg/ml Liberase (Roche), passage through an 18-gauge needle and the cells were collected by centrifugation (900 x g; 4°C). RBCs were lysed, the sample washed and the cells re-suspended. Interstitial CD45^+^ and CD45^-^ cells were then separated and the appropriate fractions enriched using an autoMACS cell separator after being labeled with microbeads coupled antibodies to CD45 (Miltenyi Biotec). Fractionation purity routinely exceeded 95 percent as did cell viability.

For flow cytometry (FC) analysis 0.5–1 x 10^6^ cells were counted, resuspended in 200μl FACS staining buffer (PBS, 2% BSA, 0.05% EDTA, SA 0.1%) and placed into tubes on ice. For all samples, Fc receptors were blocked using 1μg/sample Fc block for 15 minutes on ice. These cells were labeled with antibodies to CD45, CD11c and/or SiglecF as described in detail [7, 12] and as noted in the text. Thirty thousand events (live nucleated cells) for each sample were collected using an Accuri Cell Cytometry System (Ann Arbor, Michigan) and subsequently the data were analyzed with CFlow software (Ann Arbor, Michigan). This characterization is complicated by high AM autofluorescence that makes measurement of these cells, especially after CS exposure, particularly difficult. To correct for this, FC filters (attenuation filters (FL1 (CD144), FL2 (CP148), FL3 (CP164), and FL4 (CP165)) for the Accuri flow cytometer) were placed in the fluorometer to substantially eliminate non-specific autofluorescence from control samples (Methods, and [11, 12]). However, cells from CS-exposed mice retained significant autofluorescence and this limited their further characterization.

### RNA-seq

The methods to enrich epithelial cells from the lung interstitium for transcript expression analysis using RNA-Seq has been described previously [7, 11, 12]. Briefly lungs were cleared of BALF content, the trachea removed and the more distal lung lobe tissue enriched by dissection. The interstitial cells were collected and reacted with anti-CD45 antibody coupled to magnetic beads before depletion of the CD45^+^ cells using an autoMACS cell separator (Miltenyi Biotec, San Diego, CA). Isolated CD45^-^ fractions were used to prepare poly-adenylated strand-specific RNA-Seq libraries using Illumina TruSeq stranded mRNA preparation kits after confirmation of their purity (>95% epCAM^+^, <1% CD31^+^ endothelial cells) and viability (<5% 7-AAD (viability stain) positive as before [7, 11, 12]). To focus on identification of α7 participation and calcium relevant signaling events in the chronic CS response we used an approach similar to that reported previously that reduces false-positive signals such as those created by minor transcripts [7] and permits a more focused analysis on regulatory and metabolic genes. Briefly, RNA-Seq reads were aligned to the mouse genome (UCSC Genome; Assembly NCBI37/mm9) using STAR [14] and CDS read counts were then extracted from the bam files using the *htseq-count* script and CDS annotations from mouse GENCODE reference release M1 (NCBIM37) [15]. Based on all samples (male, female, genotype and CS exposed or not exposed) for each gene an average CDS value was derived and those with less than 200 counts were removed from further analysis as were other transcripts such as those encoding immunoglobulins, histocompatibility genes, genes without an assigned gene name (GM prefix class of Ensemble annotations) and eukaryote ribosome subunits. For this analysis we averaged male and female results from normalized reads per million and produced ratio-changes to better define those transcripts exhibiting a CS or α7 genotype response for the 7183 transcripts that remained. As previously discussed [7], this relatively stringent cut-off improves the detection of changes of biological relevance to cell functions when using detection with database resources such as GeneMANIA [16], STRING [17], Panther [18] and PASTAA [19]. Also, as previously reported [7], the expression of key inflammatory genes were examined using TaqMan real-time quantitative PCR. Similar to these reports these qPCR checks confirmed the results collected using CDS-relative quantitation (not shown). The data used in this study are deposited as an NCBI GEO Submission (GSE105089).

### Lung histology

For histological examination a total of 5 α7^G^ and 6–8 α7^E260A:G^ from each treatment group (+/-CS) lungs were prepared for histological examination using paraformaldehyde inflation as described previously [7]. Perfusion fixation was used to better preserve the relative volume of the tissue and approximates the sizes of structures as they would appear during inhalation. The larger lobe (right lung) was removed and sectioned (5 microns) longitudinally. Lungs were coded and submitted to Histology Tech Services (Gainesville, FL) for sectioning and staining (Hematoxylin and Eosin (HE), Masson’s Trichrome and Sirius Red). Slides were returned, photographed and analyzed for the parameters noted in the text prior to revealing their identity. The fractal deviation of the bronchial lining from smooth [20–22] was measured in sections stained alternatively with hematoxylin and eosin or Masson’s trichrome staining, respectively. Photos of proximal and distal lung containing sections (N = 6–9 sections per staining method for each mouse mice) were captured and deviation from smooth reordered for each bronchiole cross-section present and summed per animal before deriving an overall genotype/treatment result. Statistical tests (Student’s t-test and ANOVA and) were performed using the Excel or GraphPad InStat3 programs and significance was assigned at p<0.05. Immunohistochemistry to reveal the club-cell specific antigen, CC10, was done using peroxide staining methods as described previously [7]. Antibodies for peroxide immunohistochemistry were for CC10 (Clara cell 10, 1:500, rabbit, Millipore) and donkey secondary anti-rabbit antibodies coupled to peroxide were obtained from Jackson ImmunoResearch).

## Results

The nicotinic-α7 receptor interaction is a central component to how nicotine, the addictive agent of CS, imparts an anti-inflammatory effect in peripheral tissues. Previously we demonstrated the expression of α7 transcripts by alveolar macrophages (AM) and in the epithelium, club and ATII cells. All of these cells exhibit a response to the inflammagen, LPS that is modulated through α7-signaling mechanisms. The goal of this study was to extend these results and assess the impact of α7 signaling in the mouse lung after CS exposure. To achieve this goal, the status of AM and transcriptional responses by epithelial cells was measured in mice (male and female) of both control (α7^G^) and α7^E260A:G^ (calcium-restricted cell signaling) genotypes that were exposed to cigarette smoke (CS) 5 days per week for 4 months (Methods). Mice from two independent replicate experimental groups were used for these experiments over the course of a year.

### FC analysis and histology of lungs exposed to CS

**Fig 1:**
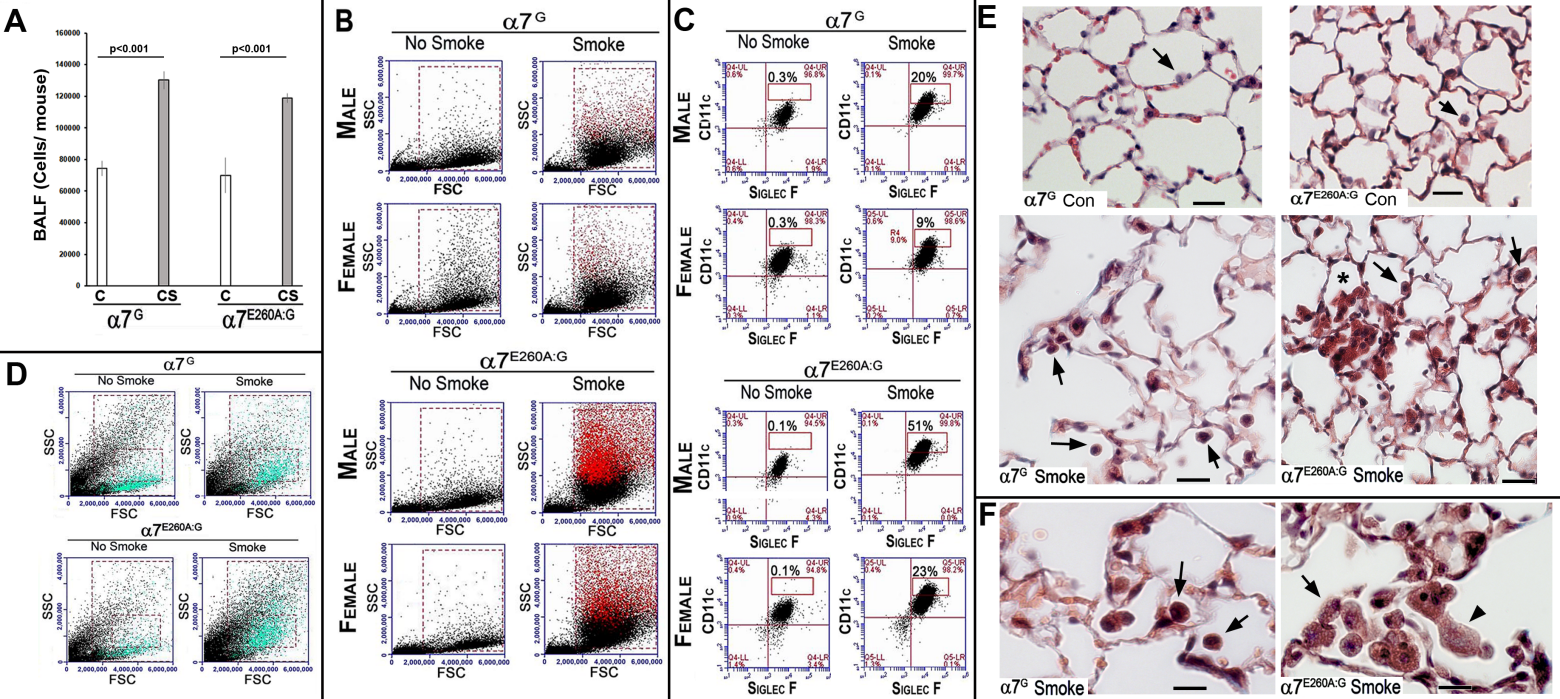
Flow cytometry of BALF and histology of lung sections prepared from α7^G^ and α7^E260A:G^. A. Average total cells recovered in the BALF as measured for each animal before and subsequent to CS exposure. Error bars = s.e.m. B. Flow cytometry results of BALF cells from the lungs of α7^G^ or α7^E260A:G^ males and females never exposed to cigarette smoke (No Smoke) or after CS (Smoked) for 4 months. Following CS the number of cells in BALF increases in both α7 genotypes, but significance (T-test) was reached (p < 0.007) only between comparisons of α7^E260A:G^ No-CS versus CS exposed (not shown). Both genders of the α7^E260A:G^ mice show significant increases in AMs following CS. For each gender the upper row of images show results from staining BALF cells with anti-CD11c and anti-SiglecF antibodies (AM are CD11c^+^/SiglecF^+^). Greater than 94% of BALF cells under all conditions are AM. The box outline in each plot represents the percent of these cells that are CD11c^+hi^ indicating a greater activation state which is only present in chronic CS BALF. The reduced relative expression in the females compared to males does not occur in all animals and is not significant (p>0.05). C. The location in scatter plots of the CD11c^+hi^ cells (boxed areas in panel A) was determined by back coloring the cells red (CFlow program and location in FSC/SSC plots). D. Flow cytometry of cells from the dissociated distal lung interstitium of both α7 genotypes following CS exposure. There is an increase in a population (blue cells) in the SSC and FSC plots after CS. As a result of very high autofluorescence, staining to identify these cells is not possible. E. Histological sections from lungs prepared from animals treated in parallel to those used for Flow analysis are shown. Lungs were prepared of the large lobe using inflation paraformaldehyde fixation and longitudinal sections were produced. The sections shown are contrasted using Masson’s trichrome stain. Examples of AM are identified by arrows. After CS AMs are discolored and they are more abundant in both α7^G^ and α7^E260A:G^ genotypes. In the α7^E260A:G^ lung these cells are commonly observed in aggregates (asterisks) and their morphology is more heterogeneous than in the α7^G^. Bar is 40 microns. F. At greater magnification, the AM cytoplasm brown discoloration and granular appearance of both genotypes after CS is evident. The AM aggregates in the α7^E260A:G^ are common and the heterogeneity of the cell morphology within these aggregates is evident. Note the highly granular appearance of the more flattened AM cells in the α7^E260A:G^ CS-exposed lungs (arrowhead) that are uncommon in the α7^G^. Bar is 20 microns.

To determine how the cell number and composition of the BALF (Fig 1) is impacted upon by CS exposure, the bronchioalveolar lavage fluid (BALF) of non-smoked (NS) and CS exposed mice was collected, the cell numbers quantitated (Fig 1A) and their identity assessed by Flow Cytometry (FC). In a normal mouse the major cell-type recovered from BALF is the AM (>95%; CD11c^+^/CD11b^-^/SiglecF^+^; [7]). The results show that in BALF of both NS and CS mice (α7^G^ and α7^E260A:G^, male and female) show that on average the total cell number in the BALF did not differ between α7 genotypes although as expected there were nearly twice as many cells recovered per CS-exposed mouse when compared to the control (Fig 1A). Also as expected Flow cytometry revealed that >95% of cells are AM (Fig 1B), but after CS the AM in the BALF of both the α7^G^ and the α7^E260A:G^ mice exhibit a population of increased CD11c staining intensity (CD11c^hi^). For the female α7^G^ mice (n = 5 animals) exposed to CS on average 9% of AM were CD11c^hi^ (compared to 0.3% CD11c^hi^ in the NS mice) and even greater CD11c^hi^ cells were present in the α7^E260A:G^ female mice (23%, n = 5 animals)(compared to 0.1% in the NS control females). For males (n = 5), CS exposed α7^G^ mice had even higher levels of CD11c^hi^ cells (20%, Fig 1B) while in male α7^E260A:G^ mice (n = 5) 51% of BALF cells were CD11c^hi^. This difference between α7^G^ and α7^E260A:G^ CS exposed mice was present in all mice. The CD11c^hi^ cells were distinct in plots of forward scatter (FSC, size) and side scatter (SSC, granularity). This is revealed when they are back-colored to distinguish those that are CD11c^hi^ (Fig 1C; red cells) in FSC versus SSC plots. In both the α7^G^ and the α7^E260A:G^ CS mice this cell population is larger and more granular. This result is markedly different from the acute response to inhaled LPS where the increase in these classes of AM inflammatory cells is greatly reduced in the α7^E260A:G^ lung, in part due to a decrease in bone marrow-derived cell infiltration to the lung [7]. Neither CD4^+^ nor CD8^+^ cells were present in large numbers of BALF from CS exposed or non-exposed mice (not shown).

The effects of CS (4 months of exposure) on mouse lung interstitial cells after removal of BALF were also measured. Unlike the more uniform AM population of the BALF, the removal of autofluorescence through application of specific filters was not adequate for clear marker-based distinctions of cell types. However, we can comfortably interpret SSC vs FSC data of distal interstitium as in Fig 1D. Most notable is that in CS exposed mice there is a cell population in the interstitium that is significantly increased in both α7^G^ and α7^E260A:G^ mice (n = 5 mice of each genotype). This is illustrated by back-coloring CD45^+^/CD11c^+^ cells (Fig 1D). Although females are shown, males did not differ significantly from these results. Statistical analysis of the quantitation of these cells, which are composed of several cells types (including dendritic cells) revealed no statistical difference between genotypes; (n = 5 mice in each group; not shown). For some cell subtypes there are markers available that are not hindered by autofluorescence (as determined with non-stained cells). These include plasmacytoid dendritic cells (DC; SiglecH^+^/CD11c^+^) or monocyte-derived DC (CD11b^+^/CD11c^+^), and there was no statistical difference in their number among gender or genotypes of differing treatment groups (not shown). We cannot reliably distinguish resident DC (CD103^+^/CD11c^+^) in the CS samples of both genotypes (not shown), however, these autofluorescent/CD11c^+^/CD103^+^ cells in both α7^G^ and α7^E260A:G^ mice are significantly increased in CS over non-exposed controls. This will require further characterization. Measurement of interstitium for CD4^+^ or CD8^+^ cells by flow cytometry were also indistinguishable between NS and CS mice in either mouse genotype or gender (not shown).

Differences between α7^G^ and α7^E260A:G^ mice following CS exposure were examined using lung histology. Cells that express α7 in the distal lung of these mice, as reported by bi-cistronic tGFP expression, are predominantly limited to AMs, club cells and ATII cells of both genotypes [7]. However, in the α7^E260A:G^ mouse altered club cell morphology and distribution and diminished ciliated cell numbers was observed. For this analysis tissue sections from the lungs of mice from each genotype of mice of control and CS treatment groups were examined in parallel to those used for flow cytometry (4–8 mice per group; see Methods). As predicted by Flow cytometry analysis of BALF, there is a notable difference in the appearance of AMs in the α7^E260A:G^ lung compared to α7^G^ controls (Fig 1E). In controls that were not exposed to CS AMs are distributed similarly between α7 genotypes and they are relatively infrequent, especially when compared to the CS-exposed lung. After CS, α7^G^ control lungs exhibit AMs more frequently and these cells appear to be generally rounded with constitutive dark staining, likely reflecting adherence or internalization of CS particulates and associated discoloring agents. However, in the α7^E260A:G^ AMs are more commonly found within the alveolar space in aggregates that are in association with the luminal alveolar cell surfaces (Fig 1E). Also evident is a dramatic range in morphologies from round (similar to the α7^G^ control) to flattened. This is particularly apparent at increased magnification (Fig 1F) where the α7^E260A:G^ AM cell aggregates exhibit a range of cell shapes that are mostly unique to this genotype (male and female did not differ; not shown). The flattened morphology also allows their highly granular cytoplasm to be revealed. These results are consistent with those of Flow cytometry analysis where cells of larger size and granularity are more evident in the CS-exposed BALF when compared to the controls.

### CS and α7-genotype lung morphology

**Fig 2:**
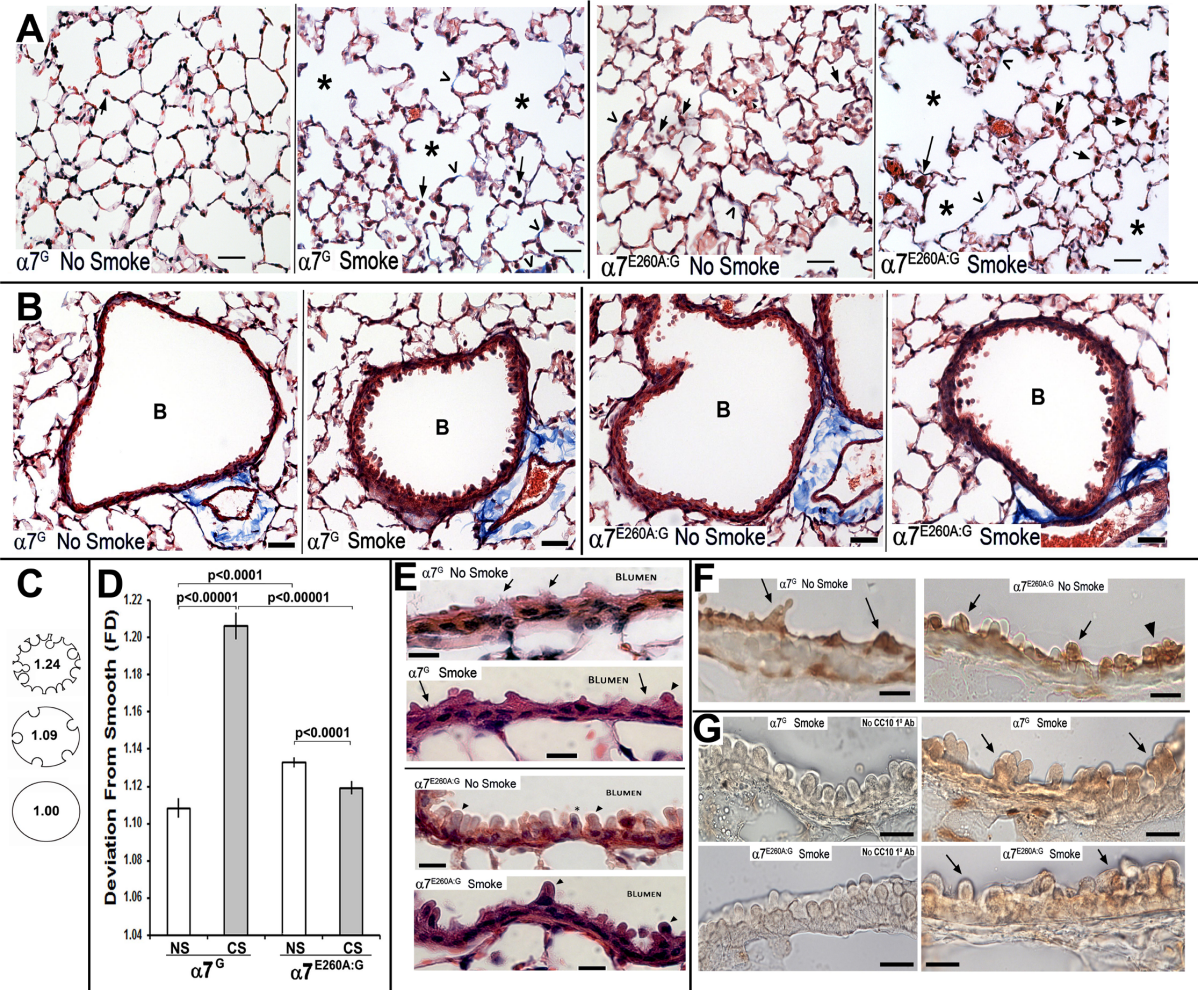
Changes to distal lung structure of both α7 genotypes with and without CS exposure. A. Images of α7^G^ and α7^E260A:G^ distal lung histological sections contrasted with Masson’s trichrome stain prepared from control (No Smoke) and CS (Smoke) exposed mice. In these representative sections the arrows point to AMs and in the α7^E260A:G^ arrow heads identify regions of increased cellularity that produce alveolar sacs of decreased size. Open arrow heads identify some of the sites of notable fibrotic deposits. Asterisks identify some enlarged alveoli in both α7 genotypes that are typical of CS exposed lungs. Bar = 40 microns. B. Representative images showing bronchioles (B) from α7^G^ and α7^E260A:G^ mice and visualized with Masson’s Trichrome staining before (No Smoke) and after CS (Smoke) as indicated (Bar = 50 microns). C. and D. The bronchiole linings were measured and quantitated for deviation from smoothness using fractal analysis (see C for example and relative scale of deviation from smoothness). The results of this analysis are plotted in panel D. Error bars reflect s.e.m. for the average deviation from all mice. E. Increased magnification of reveals bronchiole cellular hyperplasia with blebbing of cytoplasm that is particularly evident in the α7^G^ following CS (arrow heads). Some of these protrusions include cell nuclei (asterisk). The increased bronchiole club cell hyperplasia in α7^E260A:G^ mice is evident and not particularly increased by CS.; Bar = 20 microns. F. Peroxide-based immunohistochemical analysis showing signal for the definitive club cell marker, CC10, in both α7-genotypes as noted. Arrows distinguish some of the CC10-labeled club cells. Relatively large aggregates common to the α7^E260A:G^ lung are also seen (arrow head; [7]). G. Immunohistochemical analysis of a portion of the bronchial lining of CS-exposed mice as indicated and stained for CC10 using peroxidase. The panels on the left omitted primary antibody, but were otherwise handled similar to those with primary anti-CC10 on the right. Arrows indicate stained club cell blebs.

The α7^G^ and α7^E260A:G^ lung morphology in response to CS was evaluated in histological sections prepared from amice of each genotype and each CS treatment group. Overall CS exposure produced increased lung cavitation (alveolar expansion) similarly in both α7 genotypes as expected for lungs receiving this insult (Fig 2A). Several α7-genotype dependent differences in the response to CS were noted. Most notable was a change in the morphology of cells lining the bronchioli. This was striking in the α7^G^ response to CS, but less so in the α7^E260A:G^, where the morphology of the bronchiole linings was already similar to that seen in control CS mice and its response to CS exposure was more modest (Fig 2B). This apparent difference was confirmed by quantitation. To quantitate the divergence of bronchiole lining from smooth to rough (as in the α7^G^ controls), we employed an approach using fractal dimension analysis (Image Pro-Plus; and see [20–22]) to measure the deviation of the surface smoothness (Fig 2C). The results of quantitation show (Fig 2D) a highly significant difference in deviation of the α7^G^ lung bronchiole surface from smoothness following CS exposure from that of the control (P<0.0001). In contrast, the CS-exposed α7^E260A:G^ exhibited an overall increment in deviation from its genotype control that was nevertheless significant (p<0.001). The reason for this appears, at least in part, due to the increased dysplasia of the α7^E260A:G^ bronchial epithelium in the absence of CS was reported previously [7]. When compared to the α7 genotype controls, the deviation from smooth of the α7^E260A:G^ exceeds that of the α7^G^ (p<0001), but this is not worsened after CS (Fig 2D). The deviation in bronchial lining is striking at greater magnification (Fig 2E). The relatively smooth surface of the α7^G^ bronchial lining is dramatically altered by hypertrophy of airway cells that show increased cytoplasmic protrusions from the surface lining into the bronchial lumen and these are particularly evident in the α7^G^ after CS. Many of these blebs include cell nuclei. The increased irregularity to the α7^E260A:G^ cell lining are likely club cells since they express the cell-specific marker CC10 (Fig 2F and 2G; see also [7]). In the mouse lung other cells with similar morphology (such as goblet cells) are primarily located more proximal in respiratory bronchioles. The protrusions worsen in the CS-exposed α7^E260A:G^ lung although the frequency of their occurrence is greatly diminished relative to the α7^G^ CS-exposed bronchioles (Fig 2G). Also reduced in the α7^E260A:G^ is the occurrence of ciliated cells (not shown), which is consistent with our previous report [7].

### Comparison of gene expression of both α7-genotypes in CS distal lung epithelium

**Fig 3:**
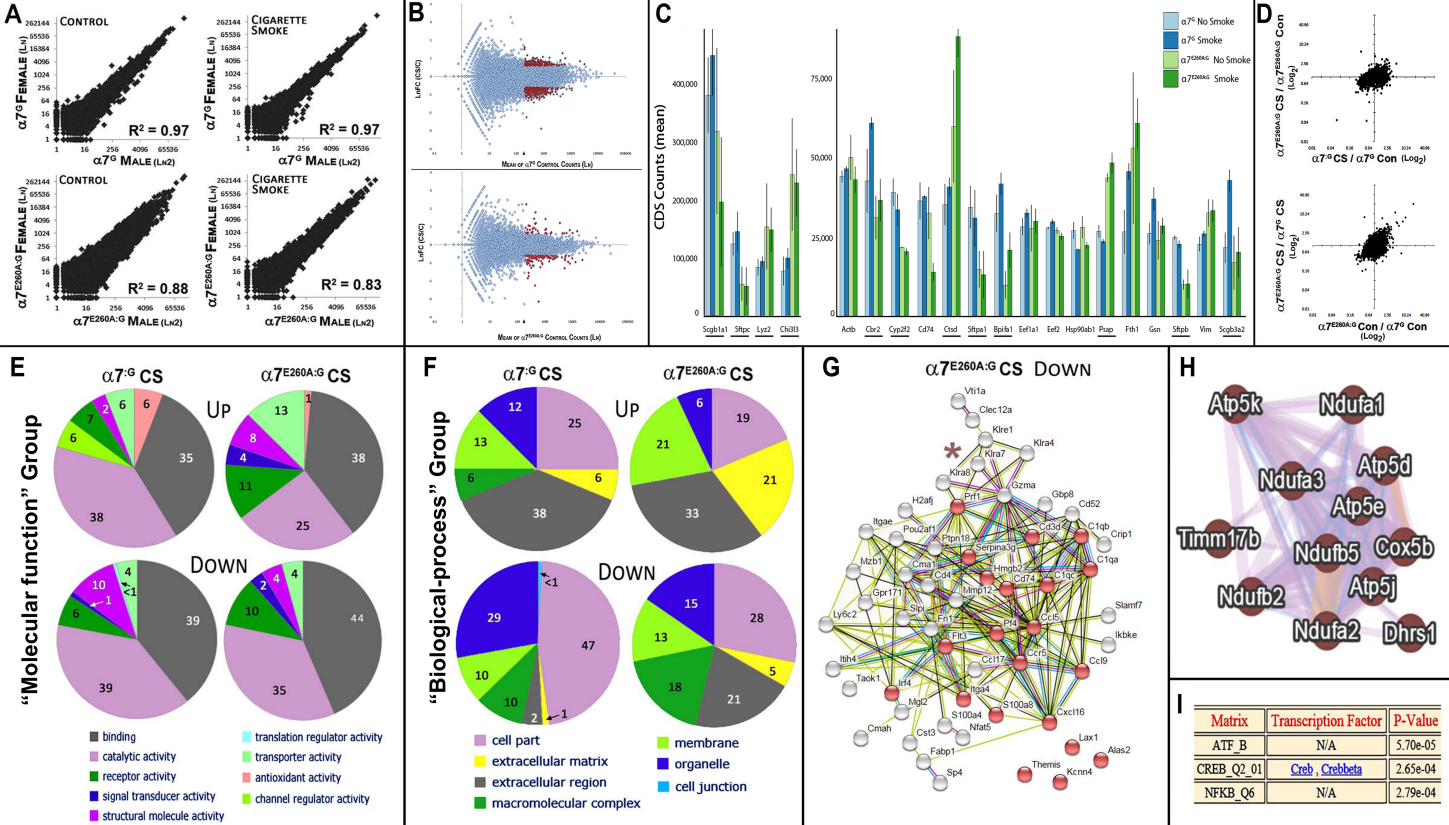
Changes in gene expression associated with CS. A. Comparison of all transcripts measured using RNA-Seq methods between males and females of each α7 genotype. CD45^-^ interstitial epithelial cells were isolated and RNA-Seq performed. Transcript data were converted to CDS values and Log_2_ plots were generated comparing α7-genotypes with and without CS exposure as indicated. Highly significant correlation values (R^2^) confirm both the absence of gender effects and the overall excellent reproducibility for this measurement. B. MA plots compare the transcriptional impact (fold-change, FC) CS has on the ratio change in expression between CS exposed and control α7^G^ epithelium (upper plot) versus those of the α7^E260A:G^ lung (lower plot). Red coloring defines transcripts that differed by >2-fold between control and CS exposure and whose CDS count was on average >200. C. Average transcript (CDS) abundance for the top 20 transcripts. Comparisons of both α7-genotypes not receiving (No Smoke) or receiving CS (Smoke) are plotted. Genes underlined differ in expression between genotypes significantly (p<0.05). The error bars reflect +/- s.e.m. D. Plots charting the overall change in expression for the remaining examined transcripts as related to either their genotype of origin (top plot) or to CS (lower plot). E. Molecular Function plots (Panther) for the top 100 responsive genes of each α7 genotype reflects similarities among gene transcript functional groupings most altered by CS (associated fold change of >2 (increased (Up); decreased (Down)). Percent values for each category are shown within each functional cluster. F. Similar to Panel D, plots reflect gene transcript assignments (Panther) to ‘Biological–process’ are shown and these exhibit greater disparity suggestive of an α7 genotype impact. Percent values are listed for each functional cluster. G. STRING analysis (Methods) of the relationship between gene products whose transcript values exhibited the most significant changes related to α7-genotype. In this case these genes are decreased by CS in the α7^E260A:G^ by >2-fold. The genes highlighted in red are related through ‘immune response’ (FDR 3.17e-8) indicative of the reduced inflammatory response seen in the α7^E260A:G^ lung epithelium. The asterisk identifies a gene group in the killer cell lectin-like receptor family (Klr xx). H. A portion of a GeneMANIA drawn diagram showing the interrelationship among genes strongly dysregulated in the α7^E260A:G^ after CS exposure. These genes are mostly located in mitochondrial respiratory complex 1 (FDR 2xe1-19). I. PASTAA analysis these suggests these genes share potential common transcriptional interactions through CREB (FDR 2.65e-4) and NF-κB (2.79e-4).

In previous studies we have demonstrated that the lung epithelium response to many inflammagens is strongly modulated by α7 [7, 11, 12]. To extend these findings of the impact by α7 coupling to transcriptional processes, we next determine the influence of CS smoke on the epithelial CD45^-^ cell fraction of the distal lungs. To do this, we isolated epithelial cells from the lungs of CS exposed mice of 4 months as above and extracted RNA for RNA-Seq analysis (Methods and [7]). The ratio between changes exceeding 2-fold in gene expression based upon average normalized read counts for each sample (Methods) that compare the CS versus Control genes are listed in S1 Table and graphic analyses of the results are summarized in Fig 3. As shown in Fig 3A, there was overall excellent agreement in gene expression between genders in their baseline gene expression and the response to CS. For α7^G^ this exceeds an R^2^ = 0.97 for both control and CS exposed experimental conditions. This value did decrease in the α7^E260A:G^ to the highly significant R^2^ of 0.88 (controls) and R^2^ = 0.83 after CS. The greater variability in the α7^E260A:G^ relative to controls is similar to previous observations of increased base-line fluctuations in the lung epithelium that lack α7-coupling to cell signaling mechanisms. Thus, in addition to confirming the correspondence in α7-associated gene expression between genders, the results also reveal the high reproducibility achieved using this method to measure transcriptome expression. When the averaged results are subjected to MA plot analysis (Fig 3B) the number of significantly altered transcripts produces an overall similar outcome although the transcript numbers impacted differ between the α7^G^ lung when compare to the α7^E260A:G^ samples (also see S1 Table). Collectively these results suggest that an α7-associated modulated component of the CS response is limited, but not absent, in the CS exposed lung.

Transcripts from a relatively small number of genes account for a large proportion of the total cellular poly(A)+ RNA pool. The 50 most abundant transcripts account for each sample more than 20 percent of all transcripts identified and the most 20 abundant transcripts over 15 percent (Table 1). For each sample group the top 20 transcripts are ranked relative to the control α7^G^ no-CS and plotted in Fig 3C. Of these 12 were significantly different in expression between α7^G^ and α7^E260A:G^ samples. The most highly expressed gene was secretoglobin, family 1A member 1 (Scgb1a1; aka, Clara cell-specific 10 kD protein, CC10). On average Scgb1a1 accounted for more than 5 percent of all α7^G^ transcripts and 3.5 percent of the α7^E260A:G^ samples. Other abundant transcripts include the ATII cell-specific surfactant protein C transcript (SftpC) followed by Lyz2 and Chi3l3. There were also genes exhibiting a substantial relative decrease in expression. Notable is the reduced expression of surfactant protein genes in the α7^E260A:G^ relative to the α7^G^ and this accompanies the increased expression of Lyz2 and Chi3l3. The reduced expression of surfactant gene transcripts by α7^E260A:G^ also extends to other ATII cell marker transcripts including Cyp2f2 as well as others including the transcript for BPI fold containing family A, member 1 (Bpifa1; aka, Splunc1), which is constitutively reduced in expression in the α7^E260A:G^ lung epithelium and whose function is associated with protecting against eosinophilic responses [23]. Finally, a particularly responsive transcript to CS that was favored in the α7^E260A:G^ lung encodes cathepsin D (Ctsd) whose function can be related to altered pro-inflammatory responses but also fibrosis [24] and low-oxygen stress conditions [25].

**Table 1:** The 20 most abundant transcripts and their respective rank relative to the α7^G^ control.

α7^G^ C*		α7^G^ CS**		α7^E260A:G^ C*		α7^E260A:G^ CS**	
Rank & Gene	RPM^1^	Rank & Gene	RPM	Rank & Gene	RPM	Rank & Gene	RPM
**1 Scgb1a1**	92635	**1 Scgb1a1**	120766	**1 Scgb1a1**	81179	**4 Chi3l3**	62005
**2 Sftpc**	30229	**2 Sftpc**	39150	**4 Chi3l3**	62133	**1 Scgb1a1**	53376
**3 Lyz2**	20367	**4 Chi3l3**	26845	**3 Lyz2**	39153	**3 Lyz2**	40112
**4 Chi3l3**	18686	**3 Lyz2**	25256	**9 Ctsd**	15297	**9 Ctsd**	23755
**5 Actb**	10804	**6 Cbr2**	16403	**2 Sftpc**	13963	**16 Fth1**	16411
**6 Cbr2**	10389	**5 Actb**	12525	**16 Fth1**	13522	**2 Sftpc**	13907
**7 Cyp2f2**	9553	**16 Fth1**	12300	**5 Actb**	12818	**21 Ctss**	13284
**8 Cd74**	8972	**20 Scgb3a2**	11543	**21 Ctss**	11889	**15 Psap**	12987
**9 Ctsd**	8582	**11 Bpifa1**	11235	**15 Psap**	11194	**5 Actb**	11613
**10 Sftpa1**	8380	**9 Ctsd**	10985	**22 B2m**	9972	**41 Ccl6**	10345
**11 Bpifa1**	7910	**8 Cd74**	10179	**41 Ccl6**	8828	**6 Cbr2**	9889
**12 Eef1a1**	6909	**17 Gsn**	9966	**19 Vim**	8344	**19 Vim**	8980
**13 Eef2**	6847	**7 Cyp2f2**	9045	**8 Cd74**	8322	**12 Eef1a1**	8095
**14 Hsp90ab1**	6608	**12 Eef1a1**	8780	**6 Cbr2**	7961	**17 Gsn**	7669
**15 Psap**	6589	**37 Scgb3a1**	8364	**29 Tmsb4x**	7461	**35 Cd9**	7656
**16 Fth1**	6464	**10 Sftpa1**	8328	**14 Hsp90ab1**	7216	**13 Eef2**	6799
**17 Gsn**	6361	**13 Eef2**	8049	**12 Eef1a1**	7100	**22 B2m**	6741
**18 Sftpb**	6101	**22 B2m**	7843	**13 Eef2**	6938	**34 Mpeg1**	6443
**19 Vim**	5568	**21 Ctss**	7171	**17 Gsn**	6164	**14 Hsp90ab1**	6017
**20 Scgb3a2**	5260	**19 Vim**	7005	**33 Flna**	5872	**24 Aldh1a1**	5966

* C = Control.

** CS = Cigarette Smoke.

^1^RPM = Average (male and female) reads per million.

When transcript values that reflect ratio changes in gene expression with and without CS (S1 Table) are plotted as in Fig 3D there is overall good agreement between the samples from different α7 genotypes. The relatively small differences are reflected by only 17 transcripts being increased in the α7^G^-CS lung epithelium >2.5-fold (41 genes > 2-fold) and 28 in the α7^E260A:G^ samples (84 genes > 2-fold). Of those decreasing by the same margin, 123 were unique to α7^G^-CS and 30 to the α7^E260A:G^-CS lung epithelium. To determine if these transcripts share common functions, an analysis using Panther functional grouping placed them into nine major subcategories in the “molecular function” (Fig 3E) and seven in “biological function” (see Fig 3F). For this analysis the top 100 responsive genes for each α7 genotype were included. The functional groups include for example those associated with activities related as ‘binding,’ ‘catalytic,’ ‘transporter’ and ‘antioxidant activity.’ Of note is the similarity in the relative proportion of gene expression that is increased by CS in both α7-genotypes. A few differences such as the greater differences in the α7^E260A:G^ lung epithelium after CS relative to the α7^G^ are noted. This includes the reduction of genes in ‘antioxidant activity’ seen in the α7^G^ lung in response to CS that is largely absent in the α7^E260A:G^ response. Also, a trend in the data towards CS-associated decreases in α7^G^ ATII-specific transcripts was noted, but this was not significant (p>0.05; not shown).

When ‘biological-process’ relationships (Fig 3F) are calculated, the α7-genotype impacts more on gene groupings in response to CS. This includes a notable increase in the expression of genes related to the extracellular matrix that is particularly strong in the CS-α7^E260A:G^, macro-molecular complexes whose expression is mostly decreased in CS-α7^E260A:G^ and organelle–associated gene transcripts such as those in mitochondria are also altered in the CS-exposed lung epithelium. The results of web-based analysis search applications (Methods) suggested these effects occur in several common functional categories. This included additional decreases in the CS-α7^E260A:G^ lung transcripts associated with ‘cell chemotaxis’ such as Ccl5, Ccr5, Ccl17, Cxcl16 and Hmgb2 (FDR 3.17e-8; Fig 3G). Also decreased are those associated with ciliary functions (FDR 1.04e-6; GeneMANIA default values), which is consistent with the decreased numbers of ciliated cells in the bronchia of this genotype [7]. Another transcript that is decreased in the CS-α7^E260A:G^ is CD74 (Fig 3G and S1 Table). This gene is expressed by ATII cells [26, 27] where it is a component of antigen processing and presentation, macrophage migration inhibitory factor signaling [28] and in some cells survival signaling through NF-κB/RelA pathways [29]. Also present is a small cluster of genes in the killer cell lectin-like receptor family (Klr(xx); Fig 3G) that are expressed by bronchial epithelium and participate in control of infections in part through regulating cytotoxic T-cell functions (e.g., [30]). In humans their expression tends to be dysregulated in those who smoke and/or have COPD [31]. Collectively, the immune-activating genes most impacted by genotype are also as a group responsive to gamma-interferon initiated innate immune processes (FDR 1.43e-4, default settings, not shown), which is consistent with known roles of α7 impact on immune function. These genes exhibit common transcriptional regulatory elements through NF-κB -associated RelA factor that was detected with a P-value of 4.6e-5 (PASTAA; not shown and Methods). This again suggests that the NF-κB/RelA controlled processes are being modulated through α7-signaling in this system.

Finally, following CS there are uniquely altered genes that exhibit close associations with the mitochondrial inner membrane (GeneMANIA Biological based setting; FDR 1.55e-6; Fig 3H). When these genes are selected and entered as a group, the FDR value increases to 2.0e-19 for highly enriched genes associated with hypoxia and those in the mitochondrial complex1 group. These same gene shifts were present in response to LPS suggesting this could be a more generalized response by the α7 genotype to inflammagen (see [7]). These genes are also found to associate with transcriptional regulatory properties through CREB-associated processes (Fig 3I; p = 5.7e-5; PASTAA results (Methods)), which is also a target of α7 cell-signaling [2, 4, 6]. Collectively this suggests that the α7^E260A:G^ response to CS includes both a change in the expression of genes important to the basic respiratory complex and also a disproportionally strong response in oxidative stress genes perhaps associated with the enhanced dysplasia in the lungs of these mice (see Fig 2 and [7]).

### The α7-genotype specific changes in CS associated fibrosis and lung cancer risk

**Fig 4:**
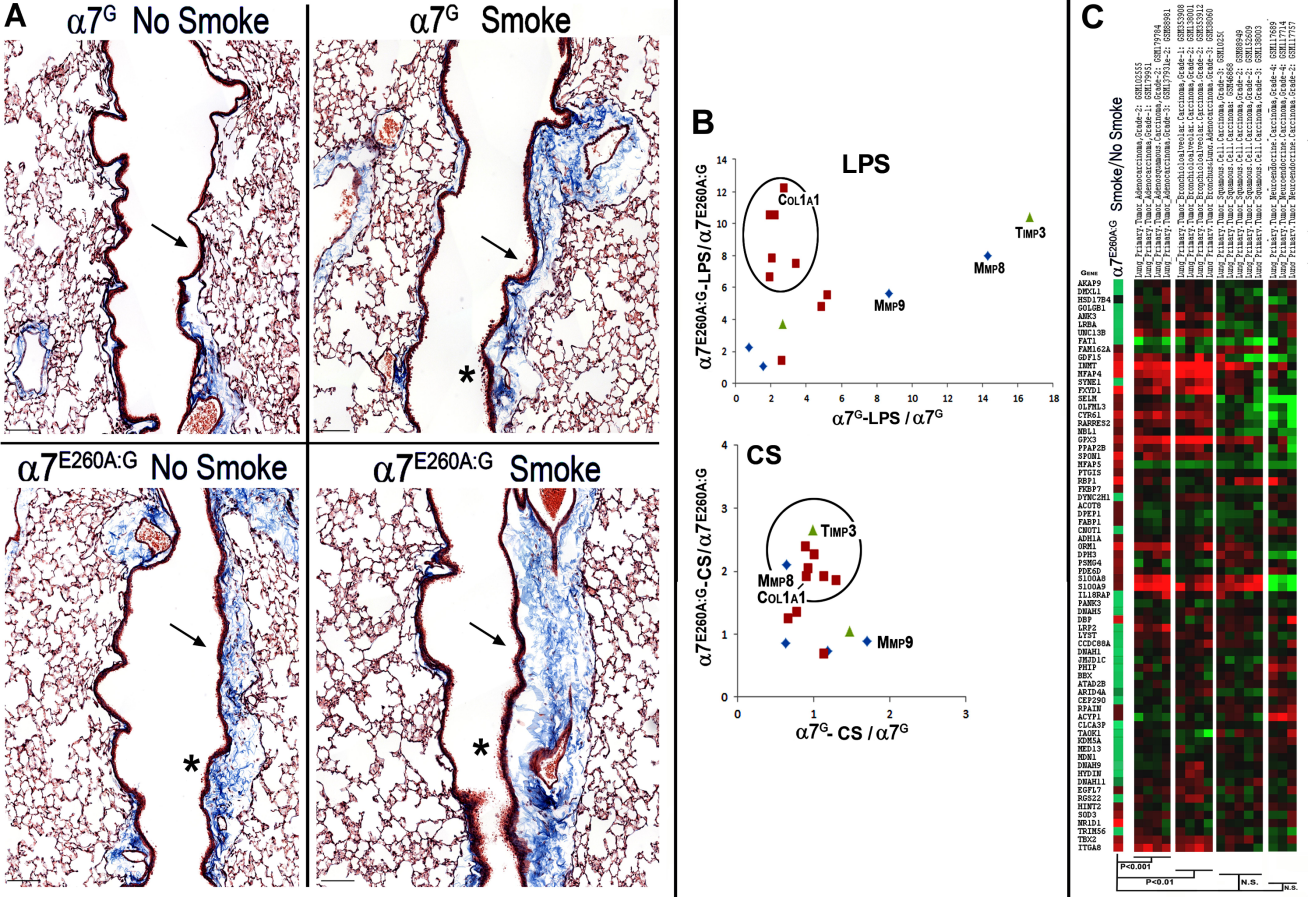
Smoking impact on fibrosis and cancer gene expression. A. Representative longitudinal sections through the large lobe of the lung of mice as indicated. Sections were visualized with Mason’s trichrome stain to emphasize fibrotic regions (blue). The regions for each genotype were photographed in the more proximal lung regions of matched major bronchial projections. Note the prominent fibrotic staining of the bronchial lining (arrows) that are particularly evident after CS, especially in the α7^G^ lung. The α7^E260A:G^ control already exhibits considerable fibrotic deposits (as before [7]) that are not increased in all animals by CS (smoke). Even at this low magnification the increased dysplasia of the bronchial lining is visible (asterisks). Bar = 50 microns. B. Plots show the relative expression of selected pro-fibrotic genes in the α7^G^ or the α7^E260A:G^ and the relative impact of either LPS (from [7]) or CS (Table 2). The genes in the circled region are strongly dysregulated in the α7^E260A:G^ after the respective treatment but not in the α7^G^ (note the scales of relative change in expression, which is more robust in the LPS treated animals). Three genes, Mmp8, Mmp9 and Timp3 are noted for being dysregulated (increased) in α7^G^ by LPS but not CS. Fibrillar collagen genes are largely unchanged although the magnitude of the dysregulated response to LPS and CS differ (Col1a1 is identified). C. Genes exhibiting the greatest changes to CS (from Table 1) are listed and assigned colors reflecting their relative change in expression after CS (Red, increased; Green Decreased). Also plotted for comparison are the same genes whose expression is reported by Metabolic gEne RApid Visualizer for the listed primary lung tumors. Spearman rank correlation values (P) for comparison between adenocarcinoma, bronchioalveolar carcinoma, squamous cell and neuroendocrine tumors are shown. NS = not significant (p>0.05).

A co-morbidity commonly associated with the late stages of chronic CS is fibrosis and increased collagen gene expression [32]. This may also be associated with promoting disease processes of COPD or idiopathic pulmonary fibrosis (IPF). Previously we reported [7] that in the α7^E260A:G^ lung epithelium there is increased fibrillar collagen gene expression as well as other genes often found elevated in fibrotic disease and their dysregulated expression was dramatically worsened after LPS exposure. This also included increased fibrosis along more proximal bronchial airways in the α7^E260A:G^ lung. This observation was examined in the context of CS in the present study. To perform histological examination of lung tissue of 5–8 mice from each CS exposed or not exposed group of both genotypes, lung sections from α7^G^ control and α7^E260A:G^ were prepared and reacted with Masson’s trichrome stain (Fig 4A). There were well-delineated fibrotic deposits along the proximal bronchia of the α7^E260A:G^ that are consistently more prominent in CS relative to the α7^G^ no-CS sections. Subsequent to CS exposure these deposits increase in both genotypes. Also there are dispersed fibrotic-like deposits at local sites in the more distal alveolar sac regions in the CS α7^G^ lung (see Fig 1D). Of note is that the enhanced fibrotic deposits that line the proximal bronchial passages of the α7^G^ lung after CS are similar to those in the α7^E260A:G^ NS lung.

To examine overlap between these data and the previously reported α7-modulated acute LPS response [7], we compared the results for the expression of potential fibrotic genes as measured using RNA-Seq. A selection of these transcripts, including a subset of fibrillar collagens (Col), metalloproteases (Mmp) and two transcripts of the tissue inhibitor of metalloproteinase gene family (Timp2 and Timp3) and their ratios between control and treatment groups are shown in Table 2. In this comparison the ratios between CDS-values for these genes from previously published RNA-Seq data of LPS treated lung tissue are included [7]. When these data are plotted, as in Fig 4B, the α7^E260A:G^ response to LPS was dominated by a disproportional increase in expression of at least 6 fibrillar collagen genes whereas changes in Mmp8, Mmp9 and Timp3 transcript expression were not α7-genotype dependent. While still elevated, the dramatic rise in α7^E260A:G^ fibrillar collagen gene expression in response to LPS inhalation was not observed after chronic CS exposure (Fig 4B). However, the expression of Mmp8, Mmp9 and Timp3 are basically unchanged from the control in the α7^G^ CS-exposed lung. Thus while there is the trend for already increased fibrillar collagen gene expression in the α7^E260A:G^ lung epithelium to be increased further after chronic CS-exposure, the impact by CS on the expression of genes in the α7^G^-genotype associated pattern seen with LPS was not observed. This suggests that the processes leading to fibrotic deposits of the CS-α7^G^, are likely different between the α7 genotypes and are highly dependent on the inflammatory challenge. Further, if α7 signaling is dysfunctional, the increased fibrillar collagen mechanisms modulated through this receptor (e.g., increased Col1a1 expression; [33]) tend to dominate the response and alter the predisposing responses towards different inflammagens.

**Table 2:** Fold-change in expression of pro-fibrotic genes between α7 genotypes and CS versus LPS.

Gene	G* CS/C	E** CS/C	G*^/^^@^ LPS/C	E**^/^^@^ LPS/C
**Col1a1**	0.91	1.92	2.75	12.20
**Col1a2**	1.01	2.27	1.94	10.51
**Col3a1**	0.89	2.39	2.24	10.52
**Col4a1**	0.67	1.20	4.87	4.78
**Col4a2**	0.79	1.34	5.23	5.52
**Col4a3bp**	0.69	1.15	2.64	1.42
**Col5a2**	0.94	2.03	1.94	6.63
**Col6a1**	1.30	1.86	3.41	7.47
**Col6a2**	1.14	1.91	2.08	7.82
**Col6a3**	1.14	0.69	1.30	4.64
**Col14a1**	1.02	2.90	2.00	7.26
**Mmp2**	1.16	2.00	3.94	7.13
**Mmp3**	1.37	2.56	4.25	11.30
**Mmp8**	1.18	0.74	14.31	8.01
**Mmp9**	1.70	0.89	8.70	5.65
**Mmp12**	0.49	0.10	0.10	0.06
**Mmp15**	0.64	2.11	0.76	2.28
**Mmp19**	0.63	0.86	1.57	1.05
**Timp2**	1.47	1.05	2.68	3.73
**Timp3**	0.99	2.66	16.68	10.45

*G = α7^G^.

**E = α7^E260A:G^

@ Values are from [7].

Mice exposed to CS for chronic periods can develop tumors of either adenocarcinoma or squamous cell histopathology [34, 35]. No evidence of tumor formation was observed in mice of this study. Nevertheless, the possibility of transcriptional changes associated with pre-cancerous tumors was explored. To do this the batch adjusted gene lists altered in cancers from all tissues were obtained from Metabolic gEne RApid Visualizer (Whitehead Institute) for human carcinomas. For those genes most associated with lung cancers, we compared the shift in ratios of the same genes expressed in the α7^G^ and α7^E260A:G^ lungs before and after CS to this list. As shown in Fig 4C, there was a significant increase (Spearman Rank Correlation) in gene expression associated with the CS-α7^E260A:G^ and transcripts of primary grade 1–3 lung adenocarcinoma (p<0.001) and primary grades 1–3 bronchioalveolar carcinoma (P<0.01; [36, 37]). Alignment with grades 2–3 primary squamous cell carcinomas or grade 2–4 neuroendocrine tumors both failed to meet significance (p>0.05). These data are suggestive of a correspondence between α7-modulated cell signaling programs and changes in gene expression associated with early stage lung adenocarcinomas as has been suggested by others [37]. This again supports the intriguing possibility of how dysfunctional α7 signaling in the local lung CS-epithelial environment could impact upon subsequent changes associated with susceptibilities to tumor development.

## Discussion

The lung response to inflammatory and irritant challenges is shaped in part by the unique local environments created from different bronchial, alveolar and interstitial compartments. The alveolar spaces of the lungs are populated by alveolar macrophages (AM) and these cells strongly express α7 transcripts [7, 11, 12]. In the BALF of both control and CS exposed mice AMs are the majority (>95%) of cells. No substantial increases in cells such as CD11b^+^ macrophages, eosinophils and other granulocytes were noted although determining their identity unambiguously was limited due to autofluorescence issues. Chronic CS exposure did coincide with increased AM numbers in both α7-genotypes and an increase in the mean fluorescence intensity of CD11c^+^ (CD11c^+hi^) by these cells. However, this increase was significantly greater in α7^E260A:G^ CS exposed mice (both male and female experimental groups) than in CS α7^G^ mice. While in both α7-genotypes there was an increase in AMs of dark brown color and increased cytoplasm granularity consistent with CS particulate phagocytosis [38], when compared to control α7^G^ the distribution of α7^E260A:G^ CS exposed AMs was irregular and often in aggregated clumps. Also their morphology differed from the mostly spheroid α7^G^ AMs to include those with a more spread-out and flattened appearance that were almost exclusive to the α7^E260A:G^. This alteration to AMs in the α7^E260A:G^ CS lung was also a feature present after acute intranasal administration (i.n.) LPS [7, 11, 12] where the responses included reduced inflammatory cell recruitment and a more alternatively activated response-like transcriptional profile when compared to the robust α7^G^ LPS response. Thus, the results are supportive of an important role played by α7 in modulating the AM inflammatory response through calcium-mediated signaling pathways that differs in outcome depending upon the source of the inflammatory insult and the identity of the responding cell(s). This raises the issues of how these mice will respond to other foreign agents such as allergens and whether or not this will alter the response such as eosinophil recruitment. In particular, it will be important to determine which effects dominate when different agents are co-exposed in the presence of CS.

The lung epithelium morphological response to CS, unlike LPS [7, 11, 12], was largely independent of α7 genotype, including CS-associated cavitation reflecting alveolar fusion present in both genotypes, but there were some subtle differences. For example, dysplasia of the bronchial surfaces was particularly prominent after α7^G^ CS exposure whereas similar deviations from a more smooth bronchial surface was already present in the α7^E260A:G^ prior to CS. Also, CS exposure does not necessarily worsen bronchial dysplasia in these lungs. This is likely due to altered club cell numbers in the α7^E260A:G^ and reduced ciliated cell number. This could also contribute to altered airway reactivity [39] and conditions of initial imbalance in the stress response, repair mechanisms and alterations to remodeling outcomes. This is consistent with the α7^E260A:G^ lung exhibition of constitutively elevated pro-fibrotic gene expression that is strongly increased after i.n. LPS but not by CS. RNA-Seq results did not reveal enhancement of epithelial transcripts for interleukins or chemokines such as eotaxins that can signal other immune cell recruitment (S1 Table and not shown). Transcriptional responses by the α7^E260A:G^ lung epithelium also differed from the control α7^G^ for genes associated with hypoxia, mitochondrial complex1 and fibrosis. These changes are consistent with known α7 signaling mechanisms that are coupled through NF-κB/RelA and Creb transcriptional pathways. For example, responses to hypoxia are in part controlled through Creb-dependent mechanisms [40]. Further, the link to genes of mitochondrial complex 1, a key modulator of timing in respiratory ATP production [41], is notable in terms of weight loss by both humans and mice exposed to CS or using nicotine [42]. The possibility that an α7-nicotine-CS interaction could produce a long-term shift in mitochondrial function through constitutively altering gene expression is suggested by this result (e.g., [43]) and offers another entry point to further examining how the nicotine-α7 interaction modifies metabolic function and possibly additional phenotypes such as weight maintenance. This could also be relevant to how CS modulation of asthma and COPD disease through NF-κB offers a target of potential therapeutic value in some individuals [44].

The issues pertaining to fibrosis and a pro-fibrotic environment are of particular note. In the α7^G^ lung the post CS pro-fibrotic appearance of the lung resembles that seen in the pre-CS α7^E260A:G^. The response in the α7^E260A:G^ lung is also consistent with other findings (e.g., [13, 45]) where uncoupling α7-mediated calcium signaling pathways either in the α7 knock-out or through nicotine induced α7 receptor desensitization/inactivation as in long-term nicotine use (for discussion of nicotine as a conditional antagonist-like ligand see [2, 5, 6]) produced similar impacts on fibrotic-like responses. This is reminiscent of studies showing that AMs of altered inflammatory response are associated with mechanisms that promote pro-fibrotic responses [46] and these have been suggested as therapeutic targets for some fibrotic diseases [47]. However, here we also see shifts in the expression of genes associated with early stage adenocarcinoma and bronchioalveolar cancers in the pro-fibrotic α7^E260A:G^ lung after CS. This may be relevant to a condition in patients with combined fibrosis and COPD who are at greater risk for developing lung cancer than patients with emphysema alone [48]. Because fibrosis in the α7^E260A:G^ model is robust, occurs independently of direct trauma, and is differentially impacted upon by LPS and CS, the additional examination of mechanisms coupling α7 to inflammatory-related fibrosis, predisposition to certain cancers and the interactions between AMs and lung epithelium signaling are warranted.

## Supporting information

S1 Table Lung epithelium RNA-Seq results summary. The ratio of gene transcript exhibiting a change in expression of >2.0 fold between treatment and α7-genotype groups. For those exceeding 200 average read depth counts between lung distal epithelium samples from the α7^G^ or α7^E260A:G^ exposed to side-stream cigarette smoke. Male and female results are combined. For these data immunoglobulin, assignments of unknown genes (GM nomenclature), histocompatibility and ribosomal genes were omitted from this analysis.
(XLSX)
